# Whole-exome sequencing identifies *MYO15A* mutations as a cause of autosomal recessive nonsyndromic hearing loss in Korean families

**DOI:** 10.1186/1471-2350-14-72

**Published:** 2013-07-17

**Authors:** Hae-Mi Woo, Hong-Joon Park, Jeong-In Baek, Mi-Hyun Park, Un-Kyung Kim, Borum Sagong, Soo Kyung Koo

**Affiliations:** 1 Division of Intractable Diseases, Center for Biomedical Sciences, National Institute of Health, Chungcheongbuk-do 363-951, South Korea; 2 Soree Ear Clinic, Seoul, South Korea; 3 Department of Biology, College of Natural Sciences, Kyungpook National University, Deagu, 702-701, South Korea

**Keywords:** Hearing loss, *MYO15A*, Mutation, Whole-exome sequencing

## Abstract

**Background:**

The genetic heterogeneity of hearing loss makes genetic diagnosis expensive and time consuming using available methods. Whole-exome sequencing has recently been introduced as an alternative approach to identifying causative mutations in Mendelian disorders.

**Methods:**

To identify the hidden mutations that cause autosomal recessive nonsyndromic hearing loss (ARNSHL), we performed whole-exome sequencing of 13 unrelated Korean small families with ARNSHL who were negative for *GJB2* or *SLC26A4* mutations.

**Results:**

We found two novel compound heterozygous mutations, IVS11 + 1 and p.R2146Q, of *MYO15A* in one (SR903 family) of the 13 families with ARNSHL. In addition to these causative mutations, 13 nonsynonymous variants, including variants with uncertain pathogenicity (SR285 family), were identified in the coding exons of *MYO15A* from Korean exomes.

**Conclusion:**

This is the first report of *MYO15A* mutations in an East Asian population. We suggest that close attention should be paid to this gene when performing genetic testing of patients with hearing loss in East Asia. The present results also indicate that whole-exome sequencing is a valuable method for comprehensive medical diagnosis of a genetically heterogeneous recessive disease, especially in small-sized families.

## Background

Hearing loss is one of the most common sensory disorders and a heterogeneous trait in human beings [1]. More than 50% of prelingual deafness is estimated to have a genetic etiology, of which about 70% is nonsyndromic hearing loss (NSHL). Eighty percent of NSHL is autosomal recessive nonsyndromic hearing loss (ARNSHL). To date, more than 100 mapped loci have been reported, and 55 nonsyndromic hearing loss genes have been identified (http://hereditaryhearingloss.org/).

The genes that are most commonly implicated in ARNSHL are in order of frequency: *GJB2*, *SLC26A4*, *MYO15A*, *OTOF*, and *CDH23*[2]. Mutations in these genes do not occur at the same frequencies across ethnicities. Thus, the current genetic diagnosis of hearing loss is limited to common mutations in a patient’s population of origin and relies on a gene-specific Sanger sequencing approach. This method is useful in some populations. For example, in Caucasians, mutation of a single gene, *GJB2*, accounts for up to 50% of all cases of ARNSHL [3]. However, in East Asian populations, *GJB2* is responsible for a much lower percentage of deafness cases, and other genes associated with this disease remain largely unknown [4].

Mutations in *MYO15A* are among the most frequent causes of ARNSHL worldwide [2]. Forty-three mutations have been reported in *MYO15A*, most of which have been found by linkage analysis in consanguineous families from specific countries, such as Pakistan, Turkey, and Iran [5-7]. Diagnostic testing for this gene is not routinely offered owing to its large size, and the frequency and spectrum of *MYO15A* mutations in most ethnic populations are not known.

Recent advances in DNA enrichment and next-generation sequencing (NSG) technology have allowed rapid and cost-effective analysis of the causative mutations for human disorders [8]. Several studies have demonstrated the sensitivity and accuracy of the exome sequencing approach as well as the clinical utility of whole-exome sequencing [9,10]. In the present study, we applied whole-exome sequencing to 16 individuals from 13 unrelated families with ARNSHL. Of them, we report novel mutations and nonsynonymous *MYO15A* variants.

## Methods

### Patients

All affected individuals had bilateral and severe to profound hearing loss without additional symptoms, and had been diagnosed with hearing impairment at an early age. Informed consent was obtained from all participants, and this study was approved by the Institutional Review Board of the Korea National Institutes of Health (NIH). Genomic DNA was extracted from peripheral blood samples using a FlexiGene DNA extraction kit (QIAGEN, Hilden, Germany). Probands from each family were found to be negative for *GJB2* and *SLC26A4* mutations based on Sanger sequencing.

### Whole-exome sequencing

Five micrograms of DNA were captured on the SeqCap EZ Human Exome Library v2.0 (Roche/NimbleGen, Madison, WI), following the manufacturer’s protocol. We targeted all well-annotated protein-coding regions as defined by the CCDS (September 2009). Captured libraries were sequenced on the Solexa GAIIx Genome Analyzer (Illumina, San Diego, CA) with 78-bp paired-end reads, following the manufacturer’s protocol. Reads were mapped to the reference human genome (GRCh37, UCSC hg19) using BWA (http://bio-bwa.sourceforge.net/). Single-nucleotide variants (SNVs) and insertions-deletions (indels) were identified using the SAMtools (http://samtools.sourceforge.net/), based on filtered variants with a mapping quality score ≥20, and annotated using ANNOVAR (http://www.openbioinformatics.org/annovar/). Mutations identified by exome sequencing were confirmed by Sanger sequencing and genotyped with additional control individuals using TaqMan SNP Genotyping Assays (Applied Biosystems, Foster City, CA). Exome-based copy-number variation (CNV) was analyzed using the ExomeCNV programs [11].

### Sanger sequencing

PCR and direct sequencing were used to prescreen *GJB2* and *SLC26A4* and to confirm the variants in the candidate genes that had been identified by exome sequencing. We also applied direct sequencing to read the missing regions (read depth <5) of candidate genes that were identified using Tablet – Next Generation Sequence Assembly Visualization [12]. The PCR products were directly sequenced, in both the forward and reverse directions, using an ABI 3730 instrument (Applied Biosystems). Each read was aligned to the reference sequence, and nucleotide changes were identified with the Sequencer software package (GeneCodes, Ann Arbor, MI).

### *In silico* analysis

Evolutionary conservation of the sequences and structures of the proteins and nucleic acids was assessed using the ConSeq server (http://conseq.tau.ac.il/). The effect of the identified novel missense mutation was assessed using SIFT (http://sift.jcvi.org), PolyPhen-2 (http://genetics.bwh.harvard.edu/pph2/index.shtml), and MUpro (http://mupro.proteomics.ics.uci.edu/). These are automatic tools for predicting the impact of an amino acid substitution on the structure and function of a human protein. The 3D molecular structure of the MyTH4 domain was modeled using SWISS-MODEL Workplace (http://swissmodel.expasy.org/). The images were captured and rendered with the PYMOL software (http://www.pymol.org/).

## Results

### Whole-exome sequencing

We performed whole-exome sequencing of 16 individuals from 13 unrelated families with ARNSHL, and found compound heterozygous mutations in the *MYO15A* gene from the SR-903 family, as well as a novel variant in the same gene from the SR-285 family (Additional file 1: Figure S1).

A summary of the exome sequencing results for the two families is provided in Table 1. On average, 97% and 90% of the bases were covered to 1× and 10× within the targeted bases, respectively. The average number of observed variants was 55,828. To identify pathogenic variants, we filtered out polymorphisms using the Single Nucleotide Polymorphism Database dbSNP131 and the Korean exome data, which includes exome data for 30 Koreans from another study [13], and the Korean genome database, TIARA [14]. We then focused on the variants of reported nonsyndromic deafness genes.

**Table 1:** Results of whole-exome sequencing of three individuals with ARNSHL

**Parameter**	**SR-903**	**SR-903B**	**SR-285**
Total reads	66,179,458	74,672,946	65,182,498
Total yield (bp)	5,161,997,724	5,824,489,788	5,084,234,844
Mappable reads	61,470,184	68,485,482	60,376,538
Mappable yield (bp)	4,669,705,365	5,194,083,022	4,596,079,334
On-target reads	39,914,806	41,803,453	36,505,698
On-target yield (bp)	2,458,150,580	2,594,965,223	2,203,746,888
Coverage of target region (more than 10×)	91%	89%	91%
Mean read depth of targeted region	56×	59×	50×
Mean read depth of called variants	46×	50×	42×
Number of total variants	56,505	54,340	56,640
Number of coding variants	20,849	20,597	20,847
Number of missense, nonsense, splice, and indel variants	10,633	10,425	10,574
After Korean control exome* filtering	543	548	510
After dbSNP131 filtering	457	472	447
Variants in reported deafness genes	5	3	3
Shared variants	3	3	-

* Korean control exome dataset, which includes exome data for 30 Koreans from another study and the Korean genomes database, TIARA.

### Identification of causative mutations

**Figure 1:**
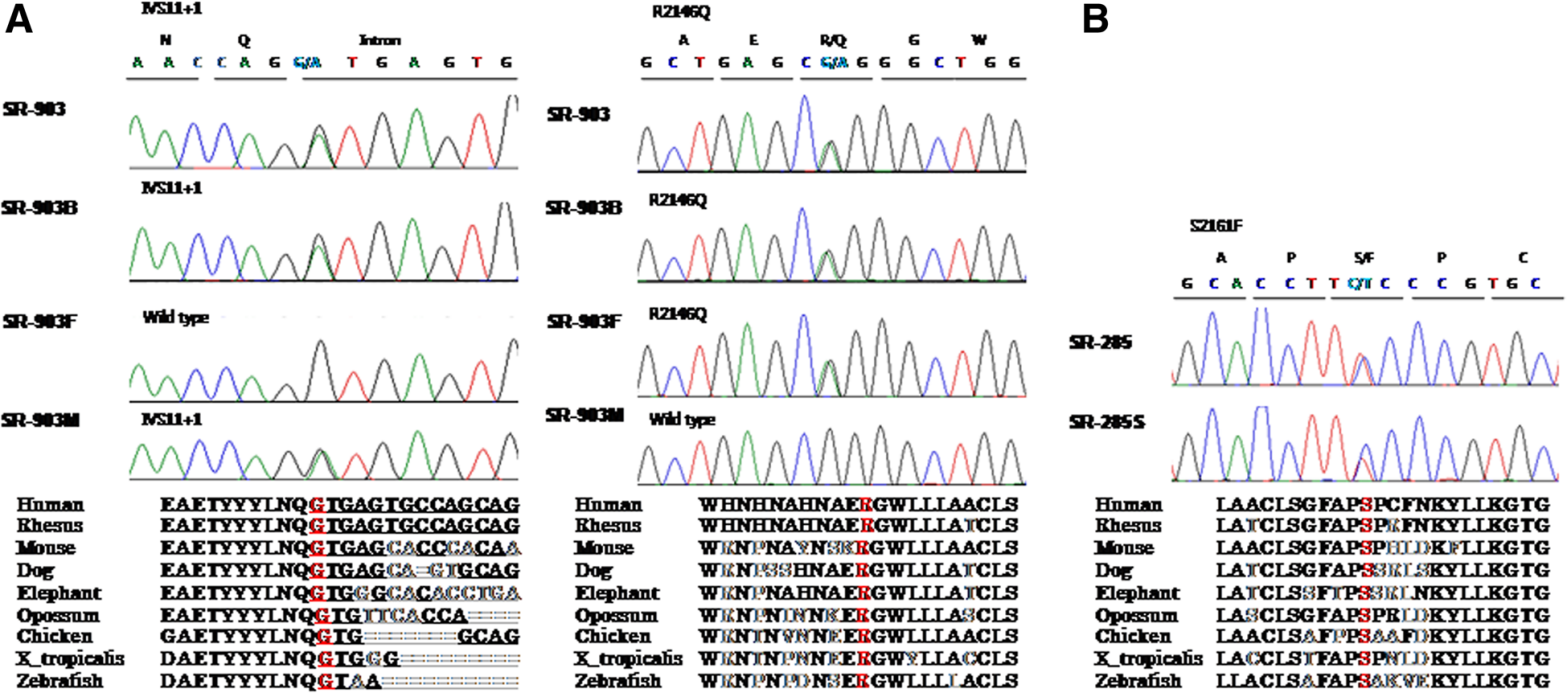
**Confirmation of novel pathogenic variants in *MYO15A*.** Causative mutations were confirmed by capillary sequencing of DNA from the families of affected patients. **(A)** In family SR-903, compound heterozygous mutations, IVS11 + 1G > A and p.R2146Q, are carried by both affected siblings, SR-903 and SR-903B. The heterozygous IVS11 + 1G > A mutation is from the mother (SR-903 M), and the other heterozygous p.R2146Q mutation is from the father (SR-903 F). **(B)** SR-285 carries the heterozygous p.S2161F mutation in *MYO15A*, and this mutation was confirmed in the affected sibling SR-285S. All the pathogenic variants occur at a highly conserved position. The corresponding DNA sequences appear in red.

In samples SR-903 and SR-903B, the numbers of remaining variants were five and three, respectively (Table 1, Additional file 2: Table S1). Of these, three variants in *MYO15A* (IVS11 + 1, p.R2146Q, and p.S3474G) were shared by both siblings. The p.S3474G variant was ruled out because it was reported in the 1000-genome database with a minor allele frequency (MAF) of 0.002, and it also appeared in dbSNP137(rs150181830). Sanger sequencing confirmed the other two variants in the affected siblings and their family. One variant, corresponding to p.R2140Q, was heterozygotic in the patient’s father (SR-903 F). The other variant, IVS11 + 1G > A, was present in the mother (SR-903 M) (Figure 1).

SR-285 carried three candidate variants of known deafness genes (p.Q445R in *GRHL2,* p.T1321S in *TECTA,* and p.S2161F in *MYO15A*); only one mutation, p.S2161F in *MYO15A*, was shared with an affected sibling (Figure 1). However, it was found to be in a heterozygous state without a second mutation. To search for other mutations, we subsequently sequenced the entire missing region (read depth <5) in all 66 exons of *MYO15A* (NM_016239). However, no additional mutation or small insertion or deletion was identified. In addition, no significantly suggestive copy-number variation (CNV) region emerged from the exome-based CNV analysis. SR-285 was found to contain another *MYO15A* coding variant, p.G1220R, which was also shared by both affected sibling but was filtered out in the initial analysis due to its appearance in the Korean exome data (Additional file 2: Table S2). In the present study, we regard this variant as a polymorphism, although given the recessive nature of this gene, it is feasible that a low frequency in the general Korean population could render it pathogenic. Unfortunately, DNA samples were not available from the parents in this family and we could not confirm pathogenicity in a segregation study.

### *In silico* analysis of mutations detected in *MYO15A*

**Figure 2:**
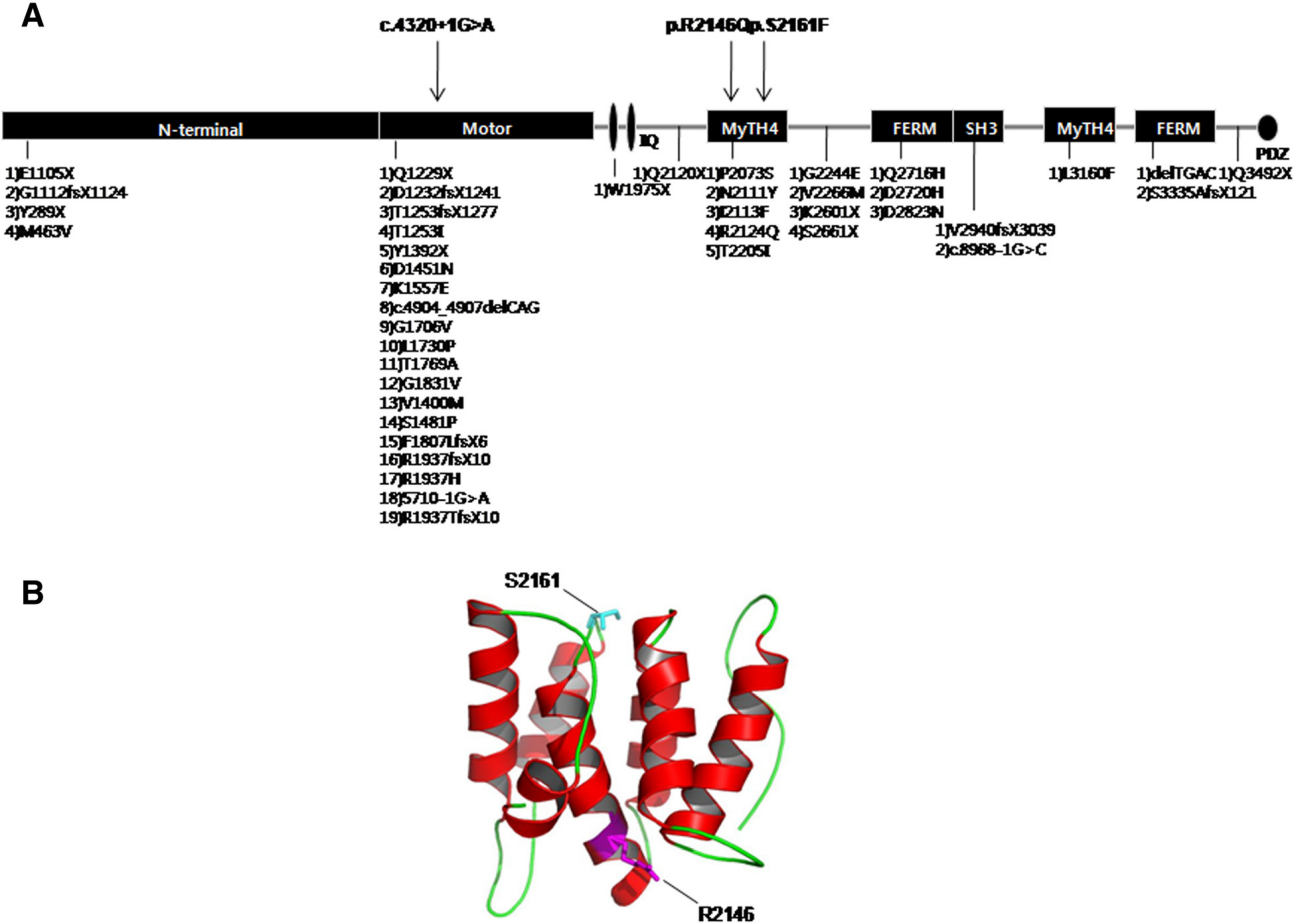
***MYO15A *gene structure and a 3D model of the MyTH4 domain. (A)** The novel MYO15A mutations reported in this study are indicated at the top of the figure, and previously reported mutations are listed at the bottom of the figure. **(B)** The MyTH4 domain comprises an alpha-helix (red) and loops (green). The locations of R2146 and S2161 are shown in magenta and cyan, respectively.

Compound heterozygous mutations in *MYO15A*, IVS11 + 1 and p.R2146Q, were found in the SR-903 family, while a heterozygous variant, p.S2161F was identified in the SR-285 family. All of the probable causative mutations were novel and were not present in 409 ethnically matched controls (Table 2). The ConSeq server revealed that all of the causative mutations were located at a well-conserved site. Residues R2146 and S2161 are both located in the first MyTH4 domain of the MYO15A protein (Figure 2A). Figure 2B presents the locations of R2146 and S2161 in a 3D model of the MyTH4 domain based on PBD entry 3pvlA. In the first variant, the substitution of an uncharged, polar glutamine for arginine results in the loss of the positive charge on the arginine side-chain (p.R2146Q). In the other variant, the polar side chain of serine is replaced with a non-polar phenylalanine side-chain (p.S2161F). Both alterations are predicted by SIFT to be deleterious. The IVS11 + 1G > A alteration is predicted to affect splicing, thereby prematurely terminates.

**Table 2:** **Characteristics of the novel *MYO15A *mutations detected in this study**

**Family**	**Genomic positions (Hg19)**	**Nucleotide change**	**Amino acid change**	**Location**	**Domain**	**Controls**	**Evolutionary conservation**	***In silico analysis***		
								**SIFT**^**1**^	**PolypPhen-2**^**2**^	**MUpro**^**3**^
**Pathologic variant**										
SR-903	Chr17:18035881	c.4320 + 1G	IVS11 + 1	Intron	Motor	0/409	Yes	-	**-**	-
SR-903	Chr17:18049349	c.G6437A	p.R2146Q	Exon 29	MyTH4	0/409	Yes	0	0.99	-0.743
**Variants with uncertain pathogenicity**										
SR-285	Chr17:18049394	c.C6482T	p.S2161F	Exon 29	MyTH4	0/409	Yes	0	1	-0.543

^1^ For SIFT, prediction of a change being deleterious (<0.05) or tolerated.

^2^ For PolyPhen-2, prediction of a change being deleterious (>0.85), possibly deleterious (0.15–0.85) or benign (<0.15).

^3^ For MUpro, prediction of a change that decreases protein stability (score <0) or increases protein stability (score >0), with a confidence score between -1 and 1.

The English in this document has been checked by at least two professional editors, both native speakers of English. For a certificate, please see:

http://www.textcheck.com/certificate/GVv7E3.

### Identification of *MYO15A* polymorphism

In addition to these causative mutations, 12 nonsynonymous variants were identified in the *MYO15A* coding exons from 17 hearing-loss patients and 30 Korean control exomes (Additional file 2: Table S2). We consider these variants to be polymorphisms because they did not cosegregate with an affected member or were found in the controls.

## Discussion

We performed whole-exome sequencing and identified novel *MYO15A* mutations in Korean families with ARNSHL. Comprehensive targeted enrichment and massive parallel sequencing have been employed for nonsyndromic hearing loss [15-17] and have focused only on the exons of all known deafness genes. However, the cost of whole-exome capture is not much more than that for only the deafness genes alone, and novel genes for hearing loss have been identified by a targeted NGS approach [18,19]. Thus, we targeted the whole exome to identify causative mutations in known deafness genes, as well as novel mutations responsible for hearing loss.

To identify pathogenic variants, we filtered out polymorphisms using the dbSNP131 and the Korean exome data. As an example, 1,462 variants in SR-903 remained after the dbSNP131 filtering. In contrast, after Korean exome filtering, 543 variants remained. This means that some variants that are commonly found in the Korean population are not present in dbSNP131. The application of Korean exome filtering more effectively removed population-specific polymorphisms. A focus on known deafness genes also greatly reduced the number of candidate variants (Table 1). In sibling samples, the identification of causative mutations is straightforward, as the mutation must be present in both siblings.

*MYO15A* (66 coding exons) encodes an unconventional myosin (myosin XVA; 3,530 amino acids) that is expressed in the cochlea [20]. This protein has important roles in the differentiation and elongation of the inner ear hair cell stereocilia [21], and it is necessary for actin organization in hair cells [22]. Mutations that cause hearing loss were first identified at the DFNB3 locus, in residents of a village in Indonesia. Since then, many mutations have been reported from Pakistan, India, Turkey, Indonesia, and Brazil [5,7]. However, the two mutations identified in the present study are novel and are the first *MYO15A* mutations reported in East Asians.

IVS11 + 1 is located in the motor domain of myosin XVA. The motor domain contains ATP- and actin-binding sites and produces the force that moves actin filaments *in vitro*[23]. Homozygous Shaker-2 (sh2) mice are deficient in myosin XVA due to a premature stop codon in the motor domain of the protein [24]. These mice exhibit profound deafness, similar to that observed in human DFNB3 patients. There are many reported mutations in this domain, and we propose that these mutations probably have deleterious effects on protein function.

Two missense mutations, p.S2161F and p.R2146Q, are located in exon 29, the first MyTH4 domain. The MyTH4 domain of myosin has been implicated in microtubule binding, as well as in actin binding at the plasma membrane [25]. There are data to suggest that the MyTH4/FERM domain in myosin VIIA is required for its localization to stereocilia tips [25-27], a process that is essential for the formation of the transmembrane actin microfilament assembly complex at the stereocilia tips. A recent study revealed that many deafness-causing mutations occur in this domain, and provided mechanistic explanations for known deafness-causing mutations in *MYO7A* MyTH4-FERM using structure-based sequence analysis [27]. In the case of myosin XVA, it has been reported that p.R2124Q and p.P2073S mutations located in the MyTH4 domain interfere with the interaction between myosin XVA and whirlin so as to prevent the formation of a complex that appears to be required for normal hearing [28]. In the present study, we identified two novel mutations, p.S2161F and p.R2146Q, which are located in the MyTH4 domain. These mutations may result in pathogenesis and deafness and may have important roles in the functions of myosin XVA. The p.R2146Q protein contains a prominent exposed hydrophobic pocket with positively charged residues and has a structure that is similar to the reported R1190 of *MYO7A* MyTH4-FERM. This region may directly bind to CEN2 at the negatively charged residues, and substitution with uncharged residues leads to an approximately 10-fold decrease in the binding affinity between MFS and CEN [27]. Therefore, we speculate that the p.R2146Q mutation cripples the inter-MyTH4-FERM interface that could lead to pathogenesis and deafness.

In SR-285, we identified two novel nonsynomous variants, p.G1220R and p.S2161F, in the *MYO15A* gene. Both of these variants were shared with the affected sibling. However, as the heterozygous variant p.G1220R was present in the Korean control exome data, we regard it as a polymorphism. Consequently, we could not identify a second deleterious mutation in this family. However, another variant, p.S2161F, was absent in 409 ethnically matched controls and was predicted to have a pathogenic effect on protein structure or function by *in silico* analysis; we would not rule out the possibility of a causative *MYO15A* gene mutation in this family. There are two possible explanations for the hearing loss observed in this family: (1) p.G1220R contributes to the phenotype of the SR-285 family and has a low frequency in the general Korean population; and (2) a pathogenic mutation exists in the *MYO15A* gene but in an exon that was not covered by our sequencing or it lies within intronic regulatory sequences. This problem highlights a limitation of exome-based analyses. Alternatively, a pathogenic mutation exists in another known gene uncovered by WES or the causative mutation is in an as-yet-unidentified hearing loss gene. Even though p.S2161F was not the causative mutation in this family, it has the potential to exert pathogenic effects on protein function in other families with hearing loss, as supported by the *in silico* analysis. Further functional assays to prove the pathogenicity of this variant would be useful to confirm the deleterious nature of this variant.

The *GJB2* and *SLC26A4* genes are frequently implicated in ARNSHL, and screening for these genes is relatively easy. Therefore, many studies have focused on only these two genes. However, a strategy for screening other hearing loss genes is difficult, and Sanger sequencing of candidate genes with multiple exons, such as *MYO15A*, is time-consuming. To screen simultaneously for each of the known deafness genes and to discover hidden mutations, we initially performed WES on 16 individuals from 13 unrelated smaller families with ARNSHL who could not be analyzed using the current genetic approach owing to insufficient genetic information following the exclusion of mutations in the *GJB2* and *SLC26A4* genes using Sanger sequencing. We identified a compound heterozygous mutation in *MYO15A* in one family and a novel variant in the same gene from another family. To our knowledge, this is the first report of *MYO15A* mutations in an East Asian population. It seems likely that there are additional ARNSHL-causing *MYO15A* mutations, as the gene is large and mutation analysis is rare, since complementary linkage analysis has not been performed in East Asian populations. The exact frequency of such mutations will need to be determined in a future larger cohort mutation analysis and close attention should be paid to this gene when performing genetic testing of patients with hearing loss in East Asia.

## Conclusions

In summary, we identified mutations in the *MYO15A* gene in a East Asian population for the first time using whole-exome sequencing. The present results indicate that whole-exome sequencing is a valuable method for comprehensive medical diagnosis and provides a valuable alternative to analyzing all known causative genes by conventional sequencing in a genetically heterogeneous disease, especially in small-sized families. In the future, the rapid discovery of hearing loss-related genes by whole-exome sequencing is expected and will provide a better understanding of the condition.

## Abbreviations

NSHL: Nonsyndromic hearing loss; ARNSHL: Autosomal recessive nonsyndromic hearing loss; NGS: Next generation sequencing.

## Competing interests

The authors do not have any conflicts of interest, financial or otherwise, to declare.

## Authors’ contributions

HMW, MHP, UKK and SKK designed the study; HMW, MHP and SKK analyzed the data and wrote the article; HJP collected the samples and examined the family information; JIB and SB performed the sequencing analyses. All authors read and approved the final manuscript.

## Pre-publication history

The pre-publication history for this paper can be accessed here:

http://www.biomedcentral.com/1471-2350/14/72/prepub

## Supplementary Material

Additional file 1: Figure S1 Pedigrees of two families with autosomal recessive nonsyndromic hearing loss. (A) Family SR-903 carries a compound heterozygous mutation in *MYO15A*, which is shared by both siblings. (B) In family SR-285, only one mutation, p.S2161F in *MYO15A*, is shared with the affected sibling. Filled symbols in each pedigree represent affected individuals. The proband is indicated by an arrow. Asterisks indicate available samples. The three individuals whose exomes were sequenced are shown in red. Click here for file

Additional file 2: Table S1 Candidate variants identified in this study. **Table S2**. Nonsynonymous mutations considered to be noncausative variations in 16 hearing-loss patients and 30 Korean exomes from another study. Click here for file
