# Supplementary figures and images for: Whole-exome sequencing identifies MYO15A mutations as a cause of autosomal recessive nonsyndromic hearing loss in Korean families
Source: BMC Med Genet. 2013 Jul 17;14:72. doi: 10.1186/1471-2350-14-72 (PMC3727941; doi:10.1186/1471-2350-14-72)

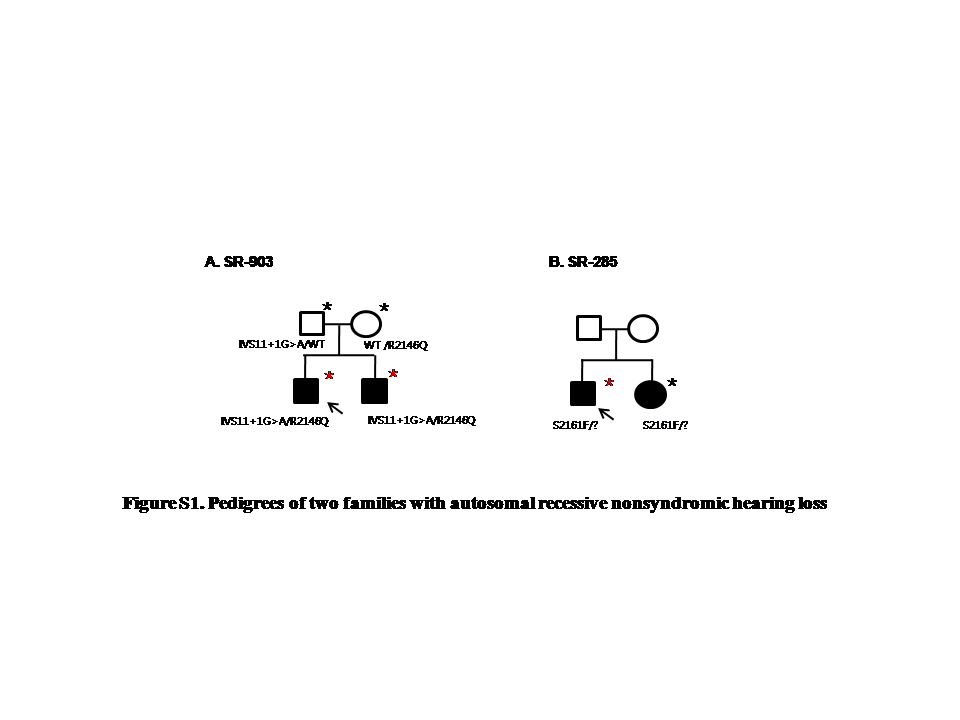

Supplement: Additional file 1: Figure S1 — Pedigrees of two families with autosomal recessive nonsyndromic hearing loss. (A) Family SR-903 carries a compound heterozygous mutation in MYO15A, which is shared by both siblings. (B) In family SR-285, only one mutation, p.S2161F in MYO15A, is shared with the affected sibling. Filled symbols in each pedigree represent affected individuals. The proband is indicated by an arrow. Asterisks indicate available samples. The three individuals whose exomes were sequenced are shown in red. [file 1471-2350-14-72-S1.tiff]
