# Supplementary material for: Whole-exome sequencing identifies MYO15A mutations as a cause of autosomal recessive nonsyndromic hearing loss in Korean families
Source: BMC Med Genet. 2013 Jul 17;14:72. doi: 10.1186/1471-2350-14-72 (PMC3727941; doi:10.1186/1471-2350-14-72)
Supplement: Additional file 2: Table S1 — Candidate variants identified in this study. Table S2. Nonsynonymous mutations considered to be noncausative variations in 16 hearing-loss patients and 30 Korean exomes from another study. [file 1471-2350-14-72-S2.docx]

**Table S1**. **Candidate variants identified in this study**

| **Sample** | **Candidate**  **genes** | **Genomic positions**  **(Hg19)** | **Gene** | **Nucleotide change** | **Protein change** | **Status** |
| --- | --- | --- | --- | --- | --- | --- |
| SR-903 | 3 | Chr4:6303782 | WFS1 | c.C2260A | p.L754I | Dominant ^A^ |
|  |  | Chr17:18035881 | MYO15A | c.4320+1G | IVS11+1 | **Causative mutation** |
|  |  | Chr17:18049349 | MYO15A | c.G6437A | p.R2146Q | **Causative mutation** |
|  |  | Chr17:18077164 | MYO15A | c.A10420G | p.S3474G | Polymorphism ^B^ |
|  |  | Chr22:38130827 | TRIOBP | c.A4484T | p.E1495B | - |
| SR-903S | 1 | Chr17:18035881 | MYO15A | c.4320+1G | IVS11+1 | **Causative mutation** |
|  |  | Chr17:18049349 | MYO15A | c.G6437A | p.R2146Q | **Causative mutation** |
|  |  | Chr17:18077164 | MYO15A | c.A10420G | p.S3474G | Polymorphism |
| SR-285 | 3 | Chr8:102643941 | GRHL2 | c.A1334G | p.Q445R | Not shared ^C^ |
|  |  | Chr11:121016686 | TECTA | c.C3962G | p.T1321S | Not shared |
|  |  | Chr17:18049394 | MYO15A | c.C6482T | p.S2161F | **Causative mutation** |

^A^ Variant was a dominantly inherited hearing loss gene, and phenotype was not consistent with hearing patients with this gene.

^B^ Variant shows polymorphisms in the 1000 genome project.

^C^ Variant was not confirmed by Sanger sequencing in another affected family member.

**Table S2**. **Nonsynonymous mutations considered to be noncausative variations in 16 hearing-loss patients and 30 Korean exomes from another study**

| **Genomic positions (hg19)** | **Exon** | **Nucleotide change** | **Amino acid  change** | **dbSNP135** | **TIARA** | **deaf_samples (16)** | **CMT_samples (30)** |
| --- | --- | --- | --- | --- | --- | --- | --- |
| 17:18023897 | 2 | c.G1783A | p.A595T, | rs2955365 | . | 3/17 | 9/30 |
| 17:18024266 | 2 | c.T2152G | p.W718G, | rs2955367 | TIARA | 1/17 | 1/30 |
| 17:18025140 | 2 | c.C3026A | p.P1009H, | rs117612144 | TIARA | . | 1/30 |
| 17:18025527 | 2 | c.A3413G | p.Q1138R, | rs76468019 | TIARA | 2/17 | 7/30 |
| 17:18027845 | 3 | c.G3658A | p.G1220R, | . | . | 1/17 (SR285) | 2/30 |
| 17:18046898 | 25 | c.T5929C | p.C1977R, | rs854777 | TIARA | 15/17 | 28/30 |
| 17:18047189 | 27 | c.G6052A | p.G2018R, | rs2272571 | TIARA | 10/17 | 28/30 |
| 17:18054497 | 38 | c.C7547T | p.A2516V, |  | TIARA | 1/17 | . |
| 17:18057167 | 42 | c.A8045T | p.Y2682F, | rs712270 | . | . | 3/30 |
| 17:18061892 | 51 | c.C9023T | p.P3008L, | . | . | . | 1/30 |
| 17:18077164 | 64 | c.A10420G | p.S3474G, | . | . | 2/17 | . |
| 17:18082164 | 65 | c.A10573G | p.S3525G, | . | TIARA | . | 1/30 |

NM_016239; TIARA is a database specifically of Korean genome data.
